# Structural and functional microbial diversity along a eutrophication gradient of interconnected lakes undergoing anthropopressure

**DOI:** 10.1038/s41598-019-47577-8

**Published:** 2019-07-31

**Authors:** Bartosz Kiersztyn, Ryszard Chróst, Tomasz Kaliński, Waldemar Siuda, Aleksandra Bukowska, Grzegorz Kowalczyk, Karolina Grabowska

**Affiliations:** Microbial Ecology and Environmental Biotechnology Department, Institute of Botany, Faculty of Biology, University of Warsaw; Biological and Chemical Research Centre, Warszawa, Poland

**Keywords:** Microbial ecology, Freshwater ecology, Microbial ecology, Biodiversity

## Abstract

We present the results of an analysis of the 16S rRNA-based taxonomical structure of bacteria together with an analysis of carbon source utilization ability using EcoPlate (Biolog, USA) metabolic fingerprinting assessment against the backdrop of physicochemical parameters in fifteen interconnected lakes. The lakes exhibit a wide spectrum of trophic gradients and undergo different intensities of anthropopressure. Sequences of V3–V4 16S rRNA genes binned by taxonomic assignment to family indicated that bacterial communities in the highly eutrophicated lakes were distinctly different from the bacterial communities in the meso-eutrophic lakes (ANOSIM r = 0.99, p = 0.0002) and were characterized by higher richness and more diverse taxonomical structure. Representatives of the *Actinobacteria*, *Proteobacteria*, *Cyanobacteria*, *Planctomycetes*, *Verrucomicrobia*, *Bacteroides* phyla predominated. In most cases their relative abundance was significantly correlated with lake trophic state. We found no similar clear relationship of community-level physiological profiling with lake trophic state. However, we found some significant links between the taxonomic and metabolic structure of the microbes in the studied lakes (Mantel’s correlation r = 0.22, p = 0.006). The carbon source utilization ability of the studied microorganisms was affected not only by the taxonomic groups present in the lakes but also by various characteristics like a high PO_4_^3−^ concentration inhibiting the utilization of phosphorylated carbon.

## Introduction

Rapid anthropogenic eutrophication of natural freshwater environments has been an increasing problem in the last few decades^1^. Eutrophication may lead to serious changes in microbial community composition, affecting the functioning of the microbial loop and thus the entire aquatic food web. Simultaneous monitoring of phylogenetic and functional (metabolic) diversity is crucial for understanding human influences on the microbial community through the acceleration of eutrophication processes. Over the past 20 years, many studies have been carried out on the diversity of bacteria in lakes with different trophic states^2–8^. Authors often present results on geographically isolated lakes located in different regions of the world with different catchment areas and, as a consequence, different physicochemical conditions. This prevents the direct comparison of the impacts of the trophic state on the microorganisms living in these separated ecosystems. However, an example of an exception is the work of Zwirglmaier *et al*.^9^, which described the seasonal changes in bacterial diversity in 19 connected pre-Alpine, hard water, alkaline lakes based on pyrosequencing of 16S rRNA genes. Nevertheless, despite the years of research focusing on lake bacteria taxonomic composition, there is relatively little information regarding the connection of the phylogenetic structure of bacteria with their metabolic functions^10^. Metatranscriptomics provides new possibilities for the analysis of potentially active metabolic pathways; nevertheless, it does not allow the determination of the actual rates of specific metabolic processes. For example, it does not directly take into account the competitive inhibition of previously produced enzymes or the influence of environmental conditions (temperature, pH, oxido-redox potential) on metabolic pathways. Therefore, it seems to be important to look for a link between the phylogenetic potential of microorganisms and the various directly measured metabolic process indicators. Using massive parallel sequencing of 16S rRNA genes^11^ together with metabolic fingerprinting, based on, for example, Biolog EcoPlate methodology^12–17^, it is becoming possible, to some extent, to make the connection between structure and function and to answer the question’who is doing what’?, at least at the community level. Answering this question is not simple because the relationships between structure and function are complex and subject to the impact of various additional conditions^18^. For example, Dickerson and Williams^19^ showed, using Biolog EcoPlate methodology, that the metabolic profiles of microorganisms from two lakes, meso- and oligotrophic, were different from those in a eutrophic lake. At the same time, the eutrophic lake showed a larger representation of *Flavobacteriia* and *Gammaproteobacteria*, but it was impossible to create a clear picture of the relationships between microorganism phylogeny and physiology. Understanding these complex relationships and the way they are influenced by human activity disturbing the physicochemical conditions of reservoirs and their trophic status is crucial for understanding the changes in biochemical cycles as a consequence of anthropopressure.

We present an analysis of the community-level physiological profiling and phylogenetic diversity of the microbial community in 15 interconnected lakes located in the temperate climate zone of north-eastern Poland. These lakes belong to the Great Masurian Lakes System (GMLS), which was formed by ice sheets during the Pomeranian phase of the glaciation in the late Pleistocene^20^. The GMLS serves as a useful model for comparative studies of lakes with different degrees of eutrophication resulting from anthropogenic stress. In addition, the lakes in the GMLS are homogenous in terms of their time of origin, geological location, catchment characteristics and climate influence. Different levels of anthropogenic pressure on lakes during the past hundred years^21^ have resulted in varying degrees of eutrophication in the Masurian lakes. In particular, stronger anthropopressure in the southern part of the GMLS has resulted in a higher trophic state in the southern part of the GMLS compared to the northern lakes^22^. An exception is Śniardwy lake, which is located at the southern end of the system. Śniardwy lake is a shallow, large lake that, despite its location and strong anthropopressure, remains in a meso-eutrophic state. We devote special attention to this particular lake because, in some respects, it combines the features of the meso-eutrophic northern and eutrophic southern lakes in the GMLS.

In this paper, we present a parallel assessment of the physiological and functional diversity in the GMLS along a eutrophication gradient in the near-shore littoral zones of the lakes. We address two main research hypotheses: (1) the taxonomic and metabolic diversities of microorganisms depend strongly on the trophic state of the studied lakes, and (2) differences in the metabolic profiles of the microorganisms are determined by the taxonomic composition—there is a strong relationship between structure and function.

## Results

**Figure 1:**
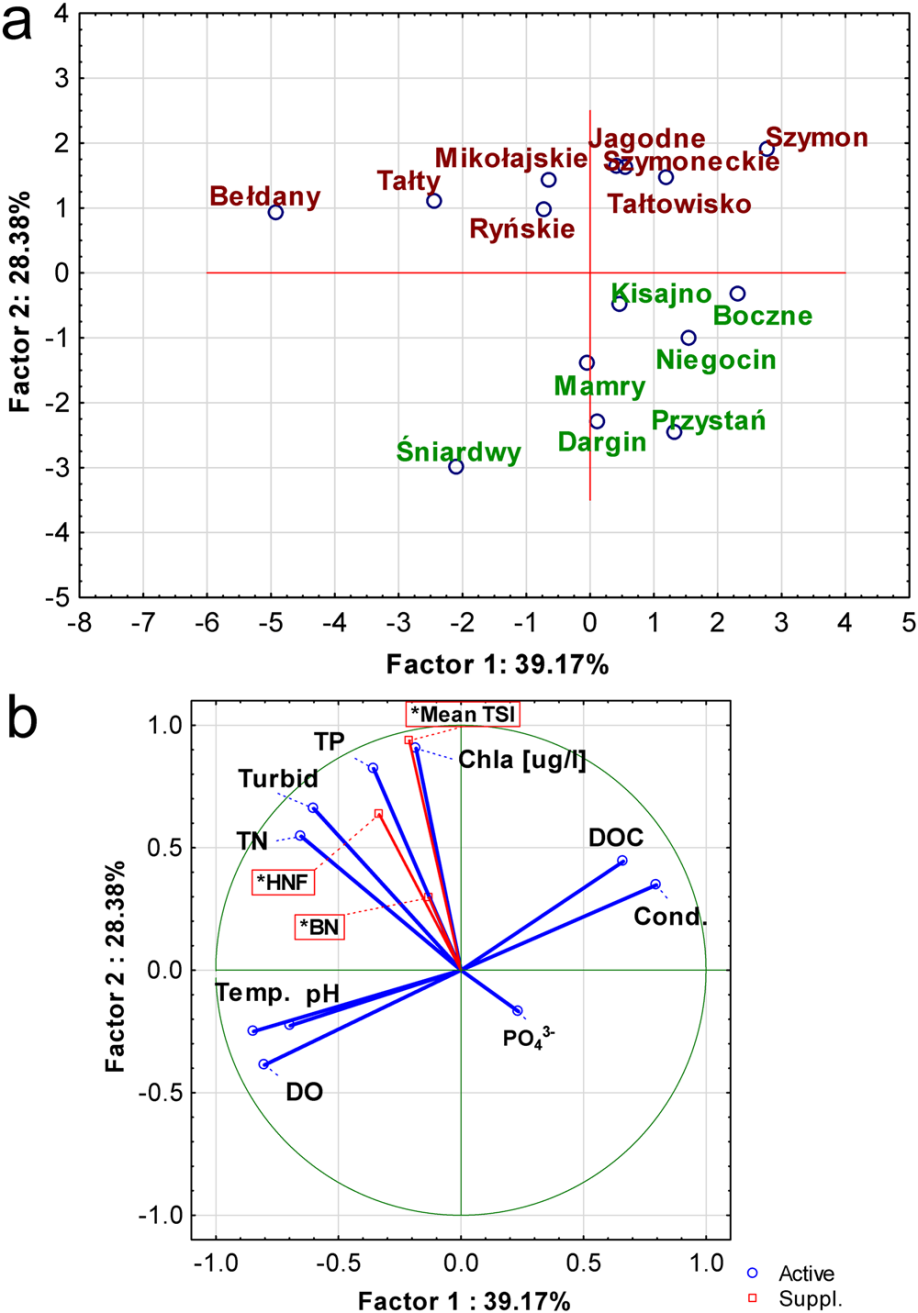
(**a**) PCA-based grouping of lakes according to physicochemical parameters. The lakes marked in red are eutrophic (E), and lakes marked in green are meso-eutrophic (ME). (**b**) Principal component analysis (PCA) results. Projection of variables on the factor planes of PCA analyses of physicochemical variables. The variables in red squares are supplementary and have no influence on the principal components of the analysis. Cond.: conductivity [µscm^−2^], DOC: dissolved organic carbon [mgL^−1^]. Chl a: chlorophyll a concentration [µgL^−1^], HNF: heterotrophic nanoflagellates [cellmL^−1^], TN: total nitrogen concentration [µgL^−1^], Turbid: nephelometric turbidity [NTU], Temp.: temperature [°C], DO: dissolved oxygen concentration [mg O_2_L^−1^], TP: total phosphorus concentration [µgL^−1^], PO_4_^3−^: orthophosphate concentration [µgL^−1^], mean TSI: mean trophic state index, BN: bacteria number [cellmL^−1^].

The TSI values calculated for the sampling sites are shown in Table 1. The studied lakes were characterized by a wide spectrum of TSI values from mesotrophy to advanced eutrophy. For further analysis, the lakes were divided into two groups according to their trophic status: meso-eutrophic (ME), consisting of seven lakes (TSI from 40 to 53) and eutrophic (E), consisting of eight lakes (TSI from 56 to 60). We assessed the characteristics of the sampling sites taking into consideration the basic physicochemical characteristics. The following parameters were measured: concentration of dissolved organic carbon (DOC), P-PO_4_^3−^, total phosphorus (TP), total nitrogen (TN), dissolved oxygen, turbidity, chlorophyll *a*, conductivity, pH, and temperature (Table 1). The results of the PCA (Fig. 1a,b) indicate that the lakes grouped according to the degree of eutrophication to E and ME were significantly different in terms of their various physicochemical conditions (Fig. 1a). Focusing on supplementary values, we found that higher TSI values were related to bacterial (LB) and heterotrophic nanoflagellate (HNF) abundances (Fig. 1b).

**Table 1:** The basic characteristics of the studied lakes.

	LAKE	Przystań	Mamry	Dargin	Kisajno	Niegocin
Area [ha]		115	2 504	3 030	1 896	2 600
Max depth [m]		22.8	43.8	37.6	25	39.7
Coordinates of sampling location		54.207241, 21.657892	54.157234, 21.723144	54.145939, 21.731939	54.042000, 21.738594	54.009215, 21.738598
Mean TSI		42 ± 2	40 ± 9	43 ± 1	53 ± 3	49 ± 3
Trophic state		ME	ME	ME	ME	ME
Temp [°C]		21.35 ± 0.06	21.68 ± 0.141	22.50 ± 0.02	21.64 ± 1.56	22.38 ± 0.74
Cond [μScm^2^]		297.00 ± 0.0	295.00 ± 0.0	302.00 ± 0.0	324.00 ± 6.0	370.00 ± 1.4
Oxygen [mgL^−1^]		9.77 ± 0.03	10.05 ± 0.03	10.19 ± 0.0	10.27 ± 1.08	10.47 ± 0.26
pH		8.38 ± 0.01	8.32 ± 0.05	8.50 ± 0.0	8.40 ± 0.09	8.45 ± 0.0
Turbidity [NTU]		0.03 ± 0.06	4.35 ± 5.3	0.30 ± 0.14	0.87 ± 0.50	1.20 ± 0.14
Chl a [µgL^−1^]		3.77 ± 0.40	0.94 ± 1.13	3.37 ± 0.57	12.26 ± 1.69	5.29 ± 0.28
TP[µgL^−1^]		15.05 ± 0.53	15.67 ± 1.34	15.67 ± 0.53	30.16 ± 1.54	26.97 ± 0.64
P-PO_4_^3−^ [µgL^−1^]		3.68 ± 0.35	3.13 ± 0.21	3.48 ± 0.62	3.58 ± 0.30	14.26 ± 0.92
TN [µgL^−1^]		367 ± 58	633 ± 115	533 ± 152	650 ± 71	600 ± 0
DOC [mgL^−1^]		8.43 ± 0.07	8.09 ± 0.07	9.11 ± 0.08	9.23 ± 0.1	11.26 ± 0.03
HNF [cellmL^−1^]		3.92 ± 0.77 × 10^3^	2.10 ± 0.76 × 10^4^	1.47 ± 0.2 × 10^4^	2.69 ± 0.44 × 10^4^	2.13 ± 032 × 10^4^
Bacteria Number [cellml^−1^]		3.9 ± 0.39 × 10^6^	5.2 ± 0.21 × 10^6^	4.2 ± 0.41 × 10^6^	4.9 ± 0.31 × 10^6^	4.9 ± 0.24 × 10^6^
	**LAKE**	**Boczne**	**Jagodne**	**Szymoneckie**	**Szymon**	**Tałtowisko**
Area [ha]		183	420	523	154	327
Max depth [m]		25	37.4	28.5	2.9	39.5
Coordinates of sampling location		53.967407, 21.758265	53.945283, 21.721688	53.918687, 21.697991	53.891046, 21.633682	53.880376, 21.560412
Mean TSI		50 ± 4	58 ± 3	59 ± 2	58 ± 1	56 ± 3
Trophic state		ME	E	E	E	E
Temp [°C]		20.95 ± 0.01	22.03 ± 0.03	21.65 ± 0.01	20.87 ± 0.01	21.48 ± 0.91
Cond [μScm^2^]		373.00 ± 0.0	354.00 ± 0.0	354.00 ± 0.0	354.50 ± 0.71	361.00 ± 5.2
Oxygen [mgL^−1^]		10.05 ± 0.01	10.08 ± 0.08	9.55 ± 0.01	7.61 ± 0.03	9.82 ± 0.16
pH		8.44 ± 0.06	8.44 ± 0.0	8.42 ± 0.01	8.02 ± 0.01	8.31 ± 0.04
Turbidity [NTU]		1.57 ± 0.31	3.77 ± 0.11	3.60 ± 0.28	2.95 ± 0.35	1.90 ± 0.1
Chl a [µgL^−1^]		7.93 ± 0.15	23.23 ± 1.56	24.07 ± 0.42	18.11 ± 0.64	19.05 ± 0.40
TP[µgL^−1^]		31.80 ± 1.52	35.19 ± 0.18	40.13 ± 0.36	37.35 ± 1.25	30.67 ± 0.71
P-PO_4_^3−^ [µgL^−1^]		6.17 ± 0.46	3.73 ± 0.21	3.43 ± 0.21	3.38 ± 0.46	2.78 ± 0.17
TN [µgL^−1^]		433 ± 58	700 ± 100	700 ± 100	667 ± 153	867 ± 153
DOC [mgL^−1^]		11.12 ± 0.29	11.99 ± 0.08	10.08 ± 0.05	10.28 ± 0.03	11.52 ± 0.11
HNF [cellmL^−1^]		2.60 ± 0.70 × 10^4^	2.54 ± 0.37 × 10^4^	3.83 ± 0.5.4 × 10^4^	1.15 ± 0.14 × 10^4^	3.93 ± 0.53 × 10^4^
Bacteria Number [cellml^−1^]		5.0 ± 0.53 × 10^6^	4.3 ± 0.24 × 10^6^	4.2 ± 0.33 × 10^6^	6.4 ± 0.21 × 10^6^	3.8 ± 0.29 × 10^6^
	**LAKE**	**Tałty**	**Ryńskie**	**Mikołajskie**	**Bełdany**	**Śniardwy**
Area [ha]		1 160	671	498	941	11 340
Max depth [m]		50.8	20.2	25.9	46	23.4
Coordinates of sampling location		53.813409, 21.567998	53.935961, 21.544402	53.801142, 21.570925	53.686344, 21.578000	53.766621, 21.842127
Mean TSI		59 ± 4	60 ± 1	59 ± 2	60 ± 1	42 ± 4
Trophic state		E	E	E	E	ME
Temp [°C]		22.66 ± 0.07	22.59 ± 0.11	21.72 ± 0.86	23.50 ± 0.11	23.58 ± 0.13
Cond [μScm^2^]		306.33 ± 0.58	309.33 ± 0.58	310.33 ± 5.0	256.00 ± 0.0	291.36 ± 1.4
Oxygen [mgL^−1^]		11.52 ± 0.50	9.90 ± 0.04	10.12 ± 0.94	12.65 ± 0.05	13.58 ± 0.4
pH		8.54 ± 0.03	8.40 ± 0.0	8.40 ± 0.11	8.63 ± 0.01	8.46 ± 0.02
Turbidity [NTU]		6.17 ± 0.38	4.23 ± 0.06	4.80 ± 3.0	7.03 ± 0.15	0.45 ± 0.11
Chl a [µgL^−1^]		12.30 ± 5.54	20.99 ± 0.68	20.86 ± 3.00	23.74 ± 0.93	2.05 ± 1.58
TP[µgL^−1^]		58.32 ± 0.64	43.42 ± 0.82	48.86 ± 0.36	45.57 ± 1.41	16.28 ± 0.31
P-PO_4_^3−^ [µgL^−1^]		4.48 ± 0.0	3.38 ± 0.17	4.48 ± 0.30	3.58 ± 0.30	3.78 ± 0.17
TN [µgL^−1^]		800 ± 58	633 ± 0	700 ± 100	1000 ± 100	700 ± 0
DOC [mgL^−1^]		9.05 ± 0.07	9.5 ± 0.02	9.15 ± 0.11	7.55 ± 0.07	8.02 ± 0.05
HNF [cellmL^−1^]		2.42 ± 0.75 × 10^4^	3.96 ± 1.2 × 10^4^	2.35 ± 0.36 × 10^4^	4.28 ± 0.41 × 10^4^	9.21 ± 2.8 × 10^3^
Bacteria Number [cellml^−1^]		5.5 ± 0.2 × 10^6^	5.6 ± 0.12 × 10^6^	5.6 ± 0.4 × 10^6^	5.2 ± 0.35 × 10^6^	4.9 ± 0.51 × 10^6^

±Are the standard deviation of mean values. Temp: temperature; Cond: special conductivity; Oxygen: Dissolved oxygen concentration; Turbidity: nephelometric turbidity; Chl a: chlorophyll a concentration; TP: total phosphorus; P-PO_4_^3−^: dissolved orthophosphates; TN: total nitrogen; DOC: dissolved organic carbon; HNF: number of heterotrophic nanoflagellates; Bacterial number: number of DAPI-stained bacteria; Trophic state E: Lake belongs to the group of eutrophic lakes; Trophic state ME: Lake belongs to the group of meso-eutrophic lakes; Mean TSI: Mean Trophic State Index calculated on the basis of the concentration of chlorophyll a, total phosphorus and the visibility of the Secchi disc.

**Figure 2:**
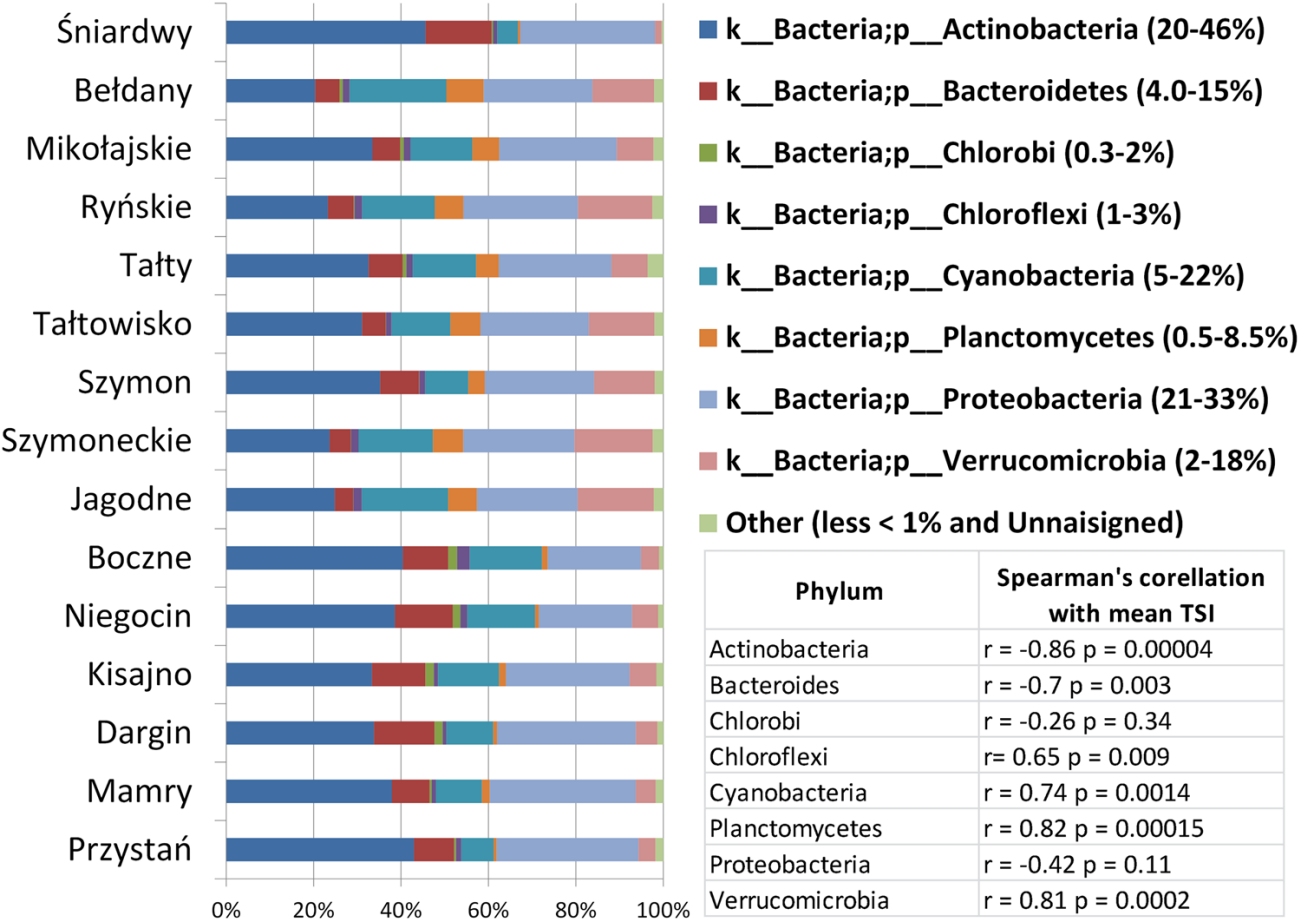
Relative sequence abundance of dominant bacterial phyla associated with different lakes. Only phyla whose relative share exceeded 1% were taken into account as individual units. Spearman correlations between the TSI and microorganisms belonging to the dominant phyla were found.

The percentages of the dominant phyla of the domain *Bacteria* in each lake are shown in Fig. 2. Only the phyla whose relative percentage exceeded 1% were taken into account as individual units. We found three dominant phyla - *Actinobacteria*, *Proteobacteria* and *Cyanobacteria*. The most abundant phylum was *Actinobacteria*, contributing from 20% in highly eutrophicated lake Bełdany to more than 40% in less eutrophicated lakes (Przystań, Śniardwy, Boczne). We found a significant positive correlation between mean TSI values and the relative abundance of the phyla *Planctomycetes*, *Verrucomicrobia*, *Cyanobacteria* and *Chloroflexi* and a significant negative correlation between TSI and microorganisms belonging to the phyla *Actinobacteria* and *Bacteroides*. This indicated that the dominant taxon might be affected by water quality parameters related to the trophic state of the sampling area.

**Figure 3:**
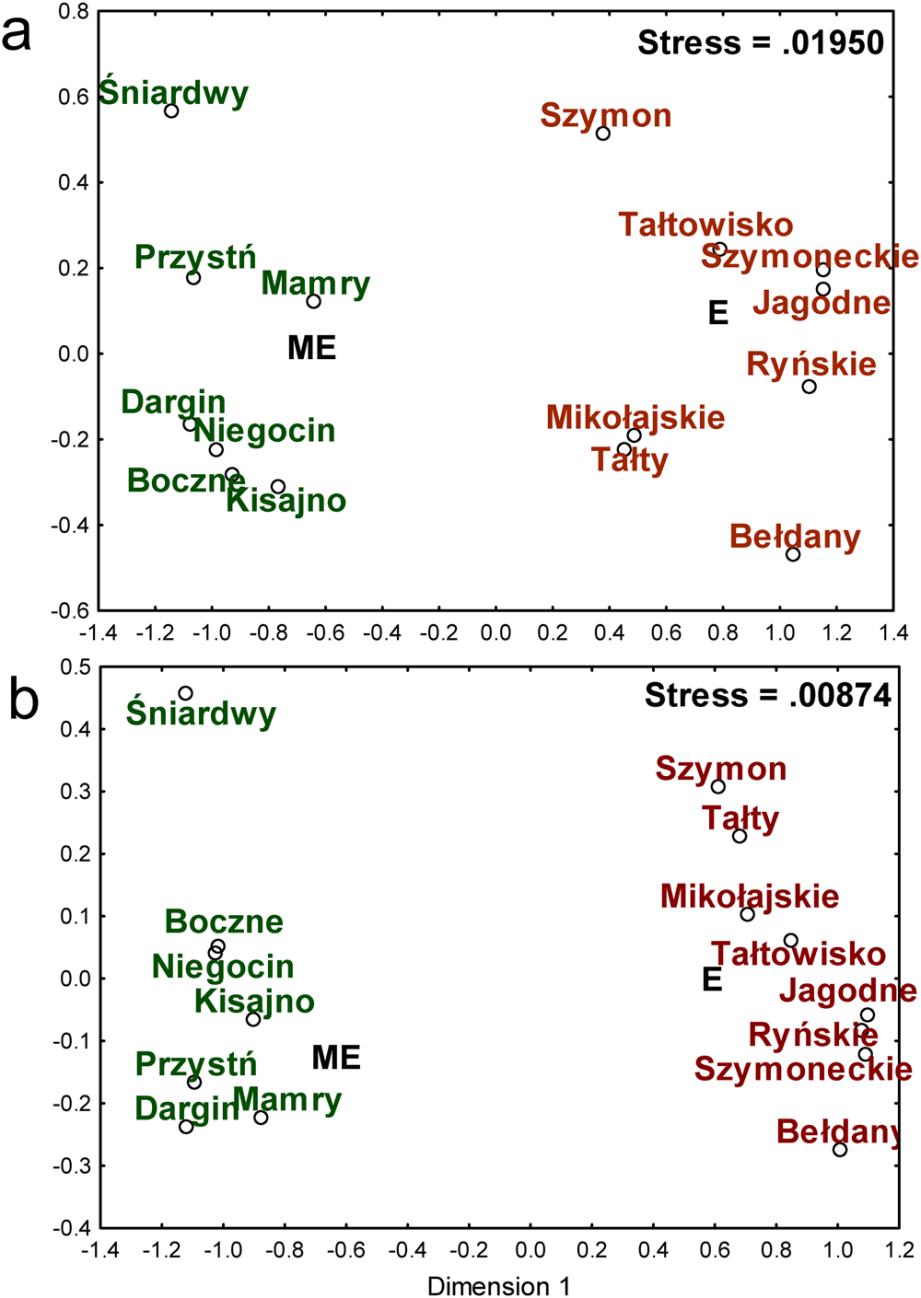
(**a**) The graphical results of the Bray-Curtis-based NMDS analysis of sequence data, binned by taxonomic assignment to family. The whole domain Bacteria was taken into consideration. The lakes marked in red are eutrophic (E), and lakes marked in green are meso-eutrophic (ME). (**b**) The graphical results of the Bray-Curtis-based NMDS analysis of sequence data, binned by taxonomic assignment to family, after excluding 25 families belonging to phylum Cyanobacteria from the analysis. The lakes marked in red are eutrophic (E), and lakes marked in green are meso-eutrophic (ME).

The Bray-Curtis-based NMDS analysis of sequence data, binned by taxonomic assignment to family, indicated that the bacterial communities living in the eutrophic lakes (E) were significantly distinct from the bacterial communities living in the meso-eutrophic (ME) lakes (Fig. 3a). The difference between eutrophic and meso-eutrophic lakes was statistically significant (ANOSIM r = 0.99, p = 0.0002). After excluding 25 families belonging to the phylum *Cyanobacteria* from the analysis, we obtained similar results. The differences between ME and E lakes were even more clear (ANOSIM r = 0.998, p = 0.0001, lower NMDS stress, Fig. 3b). To determine which taxonomic groups are mainly responsible for the observed differences between eutrophic and meso-eutrophic lakes, the SIMPER test was used. The taxonomic groups mostly responsible for the observed differences between ME and E lakes are presented in Table 2. The microorganisms belonging to the families presented in Table 2 were responsible for more than 78% of the differences observed between eutrophic and meso-eutrophic lakes. The four bacterial families with the highest influence are the *ACK-M1* family of phylum *Actinobacteria*, *Pelagibacteraceae* of the phylum *Proteobacteria*, *Chthoniobacteraceae* of the phylum *Verrucomicrobia* and *Pseudanabaenaceae* of the phylum *Cyanobacteria*.

**Table 2:** The SIMPER test results of the contribution and cumulative percentage of families mainly responsible for the observed differences between groups of eutrophic (E; consisting of eight lakes, TSI from 56 to 60) and mesotrophic-eutrophic (ME; consisting of seven lakes, TSI from 40 to 53) lakes.

Family	Contrib. %	Cumulative %
p_Actinobacteria; c_Actinobacteria; o_Actinomycetales; f_ACK-M1	13.42	13.42
p_Proteobacteria; c_Alphaproteobacteria; o_Rickettsiales; f_Pelagibacteraceae	12.35	25.77
p_Verrucomicrobia; c_Spartobacteria; o_Chthoniobacterales; f_Chthoniobacteraceae	9.503	35.27
p_Cyanobacteria; c_Synechococcophycideae; o_Pseudanabaenales; f_Pseudanabaenaceae	5.066	40.34
p_Verrucomicrobia; c_[Methylacidiphilae]; o_Methylacidiphilales; f_LD19	4.11	44.45
p_Planctomycetes; c_Planctomycetia; o_Pirellulales; f_Pirellulaceae	3.072	47.52
p_Cyanobacteria; c_Synechococcophycideae; o_Synechococcales; f_Synechococcaceae	3.025	50.55
p_Proteobacteria; c_Betaproteobacteria; o_Burkholderiales; f_Comamonadaceae	2.648	53.19
p_Verrucomicrobia; c_Opitutae; o_Cerasicoccales; f_Cerasicoccaceae	2.52	55.71
p_Bacteroidetes; c_Flavobacteriia; o_Flavobacteriales; f_Cryomorphaceae	2.296	58.01
p_Cyanobacteria; c_; o__; f_	2.104	60.11
p_Actinobacteria; c_Actinobacteria; o_Actinomycetales; f_Mycobacteriaceae	2.043	62.16
p_Planctomycetes; c_Phycisphaerae; o_Phycisphaerales; f_	1.861	64.02
p_Bacteroidetes; c_Cytophagia; o_Cytophagales; f_Cytophagaceae	1.593	65.61
p_Bacteroidetes; c_Flavobacteriia; o_Flavobacteriales; f_Flavobacteriaceae	1.559	67.17
p_Actinobacteria; c_Acidimicrobiia; o_Acidimicrobiales; f_C111	1.476	68.65
p_Chloroflexi; c_Anaerolineae; o_Caldilineales; f_Caldilineaceae	1.417	70.06
p_Cyanobacteria; c_Oscillatoriophycideae; o_Oscillatoriales; f_Phormidiaceae	1.397	71.46
Unassigned	1.319	72.78
p_Cyanobacteria; c_Nostocophycideae; o_Nostocales; f_Nostocaceae	1.226	74
p_Chlorobi; c_OPB56; o_; f_	1.199	75.2
p_Cyanobacteria; c_Oscillatoriophycideae; o_Chroococcales; f_Microcystaceae	1.19	76.39
p_Actinobacteria; c_Actinobacteria; o_Actinomycetales; f_	1.162	77.56
p_Bacteroidetes; c_[Saprospirae]; o_[Saprospirales]; f_Chitinophagaceae	1.022	78.58

We found that at the family level, the OTU richness and diversity were positively correlated with the mean TSI of the studied lakes. A higher trophic state of lakes was generally associated with both increased taxonomical richness and a higher Simpson’s diversity index, indicating a more equal distribution of detected families (Table 3).

**Table 3:** Richness (number of taxa or used carbon sources) and Simpson’s diversity index characterizing the studied lakes.

Lake	Phylogenetic Richness	Phylogenetic Simpson 1-D index	Number of used Carbon source	Used Carbon source Simpson 1-D index
Przystań	105	0.5733	30	0.937
Mamry	127	0.5889	25	0.9458
Dargin	108	0.5963	25	0.9277
Kisajno	122	0.6453	27	0.9264
Niegocin	110	0.633	24	0.9247
Boczne	129	0.6191	22	0.9283
Jagodne	140	0.6951	28	0.9368
Szymoneckie	137	0.707	28	0.9391
Szymon	135	0.6719	31	0.9456
Tałtowisko	138	0.6791	30	0.9439
Tałty	132	0.6733	18	0.9126
Ryńskie	123	0.6979	25	0.9399
Mikołajskie	136	0.6702	21	0.9302
Bełdany	132	0.7058	17	0.9234
Śniardwy	90	0.555	17	0.8976
***Spearman correlation with TSI***	***r*** = ***0***.***61 p*** = ***0***.***01***	***r*** = ***0***.***91 p*** < ***0***.***0005***	***r*** = −***0***.***1 p*** = ***0***.***7***	***r*** = −***0***.***03 p*** = ***0***.***9***

In the case of taxonomic diversity at the family level, OTU richness and diversity were positively correlated with the mean TSI of the studied lakes. In the case of metabolic fingerprinting, we did not find a significant correlation between the TSI and the number of bacterial carbon sources (richness equivalent) or Simpson’s diversity index.

**Figure 4:**
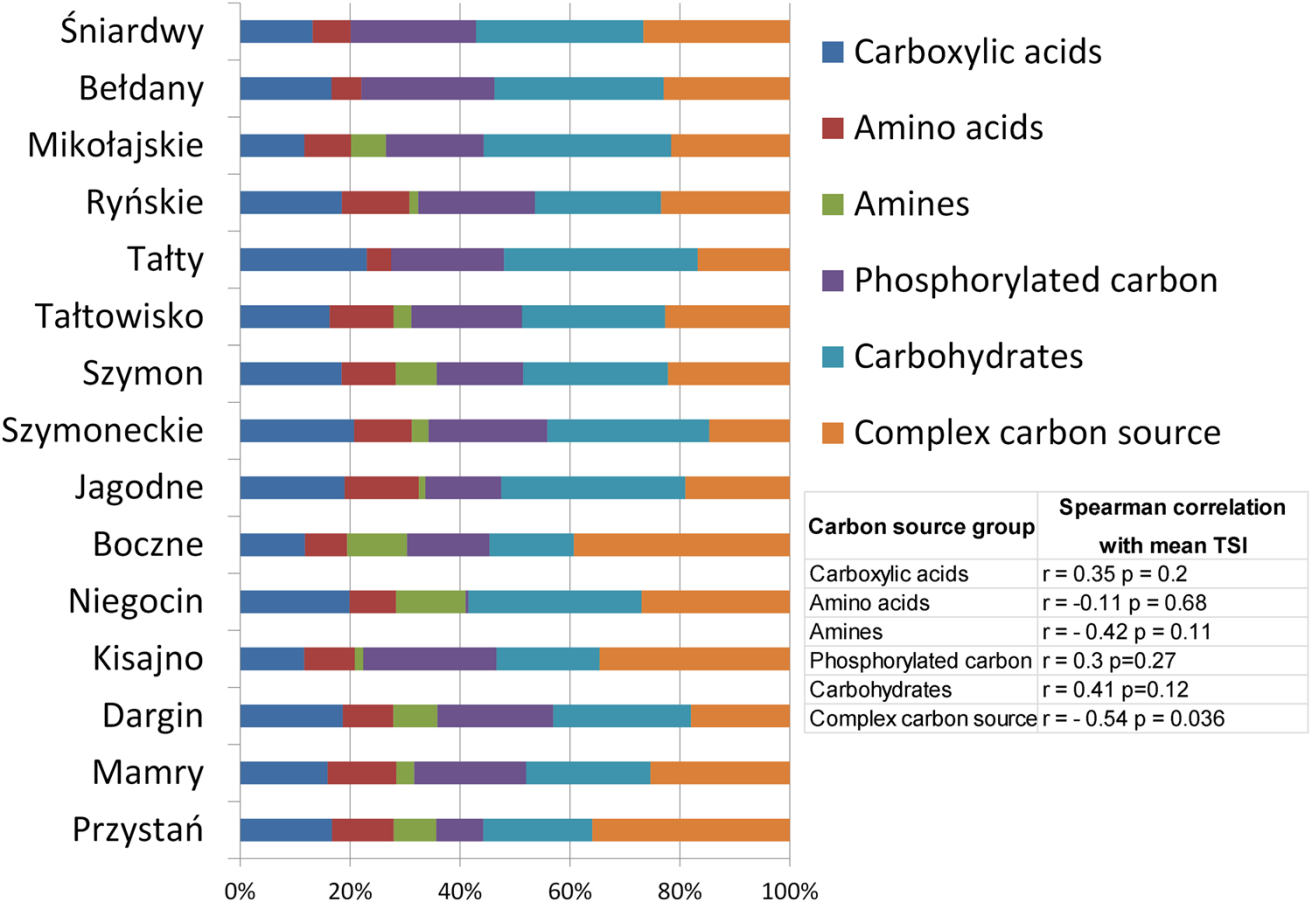
The average relative respiration of carbon sources assigned to the basic groups of compounds. Only the relative respiration rates of complex carbon sources were significantly negatively correlated with the mean TSI.

**Figure 5:**
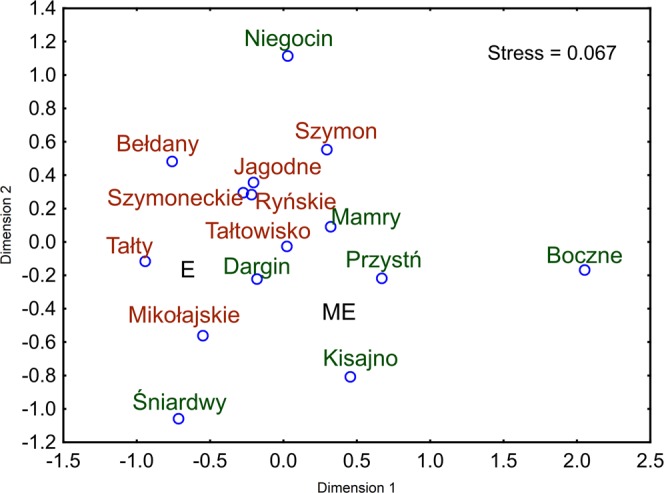
Bray-Curtis-based NMDS analysis of the relative respiration of 31 different carbon sources by bacteria in the studied lakes. The lakes marked in red are eutrophic (E), and lakes marked in green are meso-eutrophic (ME).

The average relative respiration of carbon sources assigned to the basic groups of compounds is shown in Fig. 4. We did not find a strong relationship between the respiration ability of microorganisms in association with 31 different carbon sources and the trophic status of the studied lakes. The Bray-Curtis-based NMDS analysis did not lead to an obvious grouping of the eutrophic and meso-eutrophic lakes (Fig. 5), as was the case with the taxonomic data (Fig. 3a). However, there were weak signs of grouping the lakes into eutrophic and mesotrophic based on metabolic diversity (ANOSIM r = 0.25, p = 0.003). We found that only the relative respiration rates of complex carbon sources were significantly negatively correlated with the mean TSI (r = −0.54, p = 0.03). Other carbon sources were not significantly correlated with the TSI values. It is interesting that, in the case of lake Niegocin, there is no participation of the respiration of phosphorylated carbon substrates in the total respiration of the analysed carbon sources. In Niegocin lake, we observed an exceptionally high level of P-PO_4_^3−^ (14.3 µgl^−^ L, Table 1). In general, we found a significant negative logarithmic relationship between the phosphate-carbon respiration rate and the P-PO_4_^3−^ concentration (y = −2.733ln(x) + 11.802 r^2^ = 0.8836, p < 0.05). We did not find a significant correlation between the TSI and the number of used carbon sources (richness equivalent) or Simpson’s diversity index (Table 3).

To investigate the relationship between the Bray-Curtis distance matrices created on the basis of relative phylogenetic and metabolic differences, we used the Mantel test of matrix correlations. We found that the two matrices were positively and significantly correlated (r = 0.22 p = 0.006), but the correlation was not high.

## Discussion

Carlson’s TSI index^23^ allows the numeric classification of lake trophic state. Due to the relatively high heterogeneity of lake water and the variability in measurable TSI on a yearly basis, we included the temporal TSI values characterizing the sampling sites during summer stratification in our analysis (Table 1). However, the values obtained generally correspond with the TSI values characterizing the studied lakes over several years^21,24,25^. The TSI value alone provides general information about the fertility of a reservoir and allows for a general assessment of its physicochemical properties by providing information on the amount and quality of dissolved organic matter, the number of bacteria^24^ and the penetration of light into deeper parts of the lake water body, among other variables. The classification of lakes into two eutrophic and meso-eutrophic groups was reflected in the classification of lakes based on their physicochemical diversity (Fig. 1a). It is interesting that despite the geographical location of the southern lakes and the strong anthropopressure, the sampling area of Lake Śniardwy remained meso-eutrophic^22^, and its physicochemical characteristics were similar to those characterizing the northern, less eutrophicated lakes (based on the PCA). Based on the description of the studied lakes, it can be concluded that the numbers of HNF and bacteria remain positively related with characteristics strongly associated with a high trophic state, which is in accordance with the literature^24,26^. This paper poses the question of whether the phylogenetic structure of microorganisms belonging to domain *Bacteria* inhabiting the group of eutrophic lakes differs from the structure of bacteria inhabiting the meso-eutrophic lakes. The obtained data support hypothesis that the taxonomic diversities of microorganisms depend strongly on the trophic state of the studied lakes. Obviously, Illumina sequencing does not allow for a fully quantitative evaluation of the frequencies of individual taxonomic groups based on 16S rRNA genes amplification due to, for example, differing numbers of 16S rRNA coding DNA copies in individual bacterial species or some PCR amplification bias. However, the highly significant difference between the relative occurrence of bacterial families in ME and E lakes (ANOSIM r = 0.99, p = 0.0002) leads to the conclusion that the meso-eutrophic lakes were inhabited by different groups of bacteria than the eutrophic ones. Differences in the taxonomic structure of microorganisms from domain *Bacteria* in meso- and eutrophic reservoirs can partially result from differences in the presence of cyanobacteria. As widely described^27–29^ the distribution of dominant bloom-forming cyanobacteria is usually strongly related to the geology of the catchments, nutrient loading (especially the phosphorus load), temperature and the general trophic state of lakes. We also found a positive correlation between the trophic state and the frequency of *Cyanobacteria* occurrence (Fig. 2). However, we did not find that *Cyanobacteria* significantly affected the differences in the taxonomic composition of *Bacteria* in the studied lakes. The exclusion of *Cyanobacteria* did not eliminate the differences between the two groups of lakes and even made them more pronounced (higher r^2^ and lower p in the ANOSIM test). We used the Bray-Curtis distance for the NMDS grouping of the studied lakes. On the basis of the SIMPER test, we found that the differences between the E and EM lakes were mostly due to the participation of representative of families belonging to the phyla *Actinobacteria*, *Proteobacteria*, *Verrucomicrobia*, *Cyanobacteria*, and *Planctomycetes* (Table 3). The proportions of the relative abundance of bacteria at the phylum level were similar to the results obtained by Liu *et al*.^30^, who analysed the biogeography of the abundance of bacterioplankton in the lakes and reservoirs of China.

As mentioned above, we found a strong positive correlation between the TSI and *Cyanobacteria* occurrence, which has been documented for lakes by other authors^29^. Differences in the trophic state were also reflected in the dominance of other phyla. We found a statistically significant negative correlation between the frequency of *Actinobacteria* and the TSI of the examined lakes and a high relative abundance of *Actinobacteria* compared to the that of the other detected phyla, particularly in the meso-eutrophic lakes. *Actinobacteria*, historically classified as mostly soil bacteria^31^, are frequently present in freshwater lentic systems^9,32^ and are often associated with oligotrophic ecosystems^3^. *Actinobacteria* is often the numerically dominant phylum in lake epilimnions^30,33^. Due to the production of additional pigments and high G + C content, they are relatively resistant to UV light, which easily penetrates deep into oligotrophic lakes^10^. *Planctomycetes* and *Verrucomicrobia*, on the other hand, showed a positive correlation with the TSI in our study. In the past, *Planctomycetes* and *Verrucomicrobia*, which are included in the Planctomycetes-Verrucomicrobia-Chlamydiae (PVC) superphylum, were underestimated due to the small number of deposited sequences in reference databases. Currently, the increase in the number of sequences of *Planctomycetes* and *Verrucomicrobia* deposited in the databases allows a better estimation of their role in aquatic ecosystems. The significant participation of *Planctomycetes* and *Verrucomicrobia* in the phyla found in our studies is in line with this trend. Recently, *Verrucomicrobia* have been shown to be ubiquitous in freshwater and exhibit a cosmopolitan distribution in lakes and rivers. Their abundance is usually between 1% and 6% of the total microbial community^34,35^. In our study, the mean participation of *Verrucomicrobia* was slightly higher and amounted to 9.5% (min. 1.5, max. 17.8). There is limited information about the physiology of *Verrucomicrobia* and *Planctomycetes*. It is known that the abundance of some members of the phylum *Verrucomicrobia* is favoured by high nutrient availabilities or cyanobacterial blooms^36,37^. The efficient use of glycolate by representatives of *Verrucomicrobia* is also noted^38^. Glycolate is secreted during photosynthesis by phytoplankton, occurring in higher numbers in eutrophic reservoirs (higher chlorophyll a concentrations). This corresponds to the positive correlation we found between the occurrence of *Verrucomicrobia* and the trophic status of the lakes. The *Proteobacteria* phylum did not show any significant correlation with the TSI because members of this phylum belong to a very physiologically diverse group, such as those in the *Alphaproteobacteria* or *Gammaproteobacteria* classes. *Alphaproteobacteria* often dominate in oligotrophic reservoirs and are known to be competitive under conditions of low nutrient/substrate availability^10^. In our study, the frequency of the occurrence of microorganisms belonging to the *Alphaproteobacteria* class showed a significant negative correlation with the TSI (Spearman correlation r = −0.7, p = 0.003). In addition, this class includes *Pelagibacteraceae*, which significantly contributed (SIMPER test, contrib. 12.3%) to the observed difference between the ME and E lakes (Table 3). On the other hand, *Gammaproteobacteria* (and among them many potentially pathogenic bacteria, such as *Aeromonas* spp. or *Legionella* spp.) usually require high concentrations of organic matter^10^. Gammaproteobacteria grew fast when nitrogen and phosphorus (elements occurring in high concentrations in eutrophic lakes) were added to enclosures^39^. Generally, *Alphaproteobacteria* (mostly *Pelagibacteraceae*, from 3% to 22% in our study) and Betaproteobacteria (for example, *Comamonadaceae*—in our study, their contribution was from 2% to 11%) have been regarded as some of the most important aquatic microorganisms on our planet. In our study, the abundance of *Pelagibacteraceae* was from 21% to 33%, which corresponded well with data obtained by other researchers examining various lake ecosystems^9,30,40^.

Biodiversity influences the way in which ecosystems function. We observed a significant positive correlation between the trophic status of lakes and the taxonomic richness of microorganisms and their even distribution. We found not only more taxa in the higher eutrophicated lakes but also higher values of Simpson’s diversity index in those lakes. This corresponds with results obtained by Jankowski *et al*.^41^, who showed that the bacterial community composition changed and was richer and more heterogeneous within lakes as the trophic status increased. As suggested by Bell *et al*.^42^, the greater diversity of bacteria in eutrophic lakes may be associated with their higher overall productivity and contribute to bacterial services. The same situation is observed in marine environments, where primary productivity drives greater bacterial richness^43^. It should be emphasized that the studied lakes were not hyper-eutrophic. Under hyper-eutrophy, biodiversity loss may be observed, especially in the case of phototrophic microorganisms. Nevertheless, as reported by Crossetti *et al*.^44^, the loss of biodiversity following trophic change is not a single dimension of a single factor but rather a result of a combination of factors related to larger levels of biomass in the reservoir.

In contrast to results obtained by Zwirglmaier *et al*.^9^, who showed a spatial distribution in the Osterseen Lake District, it seems interesting that in our study, the differences in the taxonomic structure of the *Bacteria* domain were associated with the trophic state rather than the geographical location of the lakes. We found some similarities that may result from the proximity of the sampling points (e.g., lakes Tałty and Mikołajskie), but the mesotrophic part of Śniardwy lake (to which water flows from Mikołajskie lake) has been classified to the group of northern lakes, which are distant from lake Śniardwy. This suggests that trophic characteristics rather than geographical proximity determine the taxonomic structure of bacteria in the studied ecosystem.

The situation is not as clear in the case of community-level physiological profiling. To obtain a community-level physiological profile, we used the commercially available Biolog Corporation’s EcoPlate test, enabling the creation of a metabolic fingerprint of the bacterial community. The most commonly used EcoPlate procedure is based on the colorimetric determination of the final concentration of the respiratory product after 48–76 hours. However, it has some limitations, which should be mentioned here. First, the tetrazolium chloride used in the test may lead to a selective reduction in the intensity of respiration of various groups of microorganisms after a longer incubation period. The accumulation of secondary metabolites and the decrease in the substrate concentration also contribute to this phenomenon. To limit this effect, we determined not only the final concentration of the colorimetric product of respiration but also the maximum rate of respiration (maximal increasing rate of the production of the colorimetric product of respiration) observed throughout the whole 72-hour incubation period. Differences in the obtained values for each of the substrates were used to calculate the Bray-Curtis similarity matrix for the studied lakes.

The EcoPlate-based results of metabolic profiling were not as strongly associated with the trophic state of lakes as was the case with phylogenetic classification. However, we also observed a tendency towards the separate clustering of microorganisms originating from meso-eutrophic and eutrophic lakes (ANOSIM r = 0.25, p = 0.003). A similar EcoPlate system was used by Dickerson and Williams^19^ to test whether three Florida lakes of different trophic levels could be distinguished by their functional profiles. These authors showed that the metabolic profiles of the two mesotrophic lakes were more similar to one another than to that of the eutrophic lake.

We did not find a significant correlation between the TSI and the number of carbon sources usable for microorganisms from lakes (richness) or between the TSI and Simpson’s diversity index. In other words, an increase in the trophic state did not contribute to an increase in the potential of the bacterial community to use a larger number of more diverse carbon sources. We also found no significant correlation (based on Spearman correlation coefficients) and no significant linear or non-linear relationships between richness and Simpson’s diversity indices for metabolic and taxonomic structures. Our results do not allow to strongly confirm or reject the concept of microbial functional redundancy described by Delgado‐Baquerizo *et al*.^45^ who, in model experiments, observed no functional redundancy in a majority of analyzed cases.

However, while the experimentally induced microbial biodiversity decline analyzed by Delgado‐Baquerizo *et al*.^45^ was very rapid, our studied lakes have been undergoing eutrophication for many years. The microbial communities in each lake are a part of a complex web of relationship between various components of the microbial loop. Relatively slow changes in biodiversity may have less influence on functional diversity than rapid changes, under which the lack of redundancy might be particularly dangerous to environmental functioning. The long term adaptation of microbial communities to environmental characteristics and available food sources is important for stable environmental homeostasis. For example, in eutrophicated lakes the excretion of organic matter by phytoplankton or degradation of dead phytoplankton cells might be a primary food source for chemoorganotrophic bacteria. In mesotrophic lakes the lack of intense primary production forces bacteria to use many different sources of carbon.

In our research, only in the case of complex carbon sources was there a significant negative correlation between the rate of use of this group of C compounds and the TSI. The utilization of complex carbon sources is typical for oligotrophic lakes, where low primary production forces microorganisms to use allochthonous organic matter. Such organic matter is often built with a long polymer chain with a high C:N ratio and is difficult to degrade^46^. The more efficient respiration of complex carbon sources in meso-eutrophic lakes that we observed may indicate the adaptation of microorganisms in less eutrophicated reservoirs to the degradation of this type of compound. Most of the literature is focused on pelagial zones; however, this zone of lakes may differ from the littoral zone in terms of bacterial activity and bacterial diversity^47^. Samples for our study were taken from littoral zones. The near-shore, ecotone and littoral zones of lakes are productive and potentially very diverse, where allochthonous matter, often of anthropogenic origin (including xenobiotics), may be a significant source of carbon. The inflow of this type of compound can have a significant impact on the metabolism of microorganisms that occupy the littoral zone of lakes.

Due to the high plasticity of chemoorganotrophic bacteria, their metabolic profiles are shaped by the presence of carbon sources in the environment, not just by the taxonomic composition. The presence of inhibitors may also have an impact. For example, in Lake Niegocin, a lack of phosphate-carbon utilization was observed in the EcoPlate-based metabolic profile. At the same time, abnormally high concentrations of orthophosphates (likely as a result of land inflow of anthropogenic origin) were found. Assimilation of phosphorylated carbon sources by microorganisms is not possible without their prior dephosphorylation. To acquire phosphorus from organic phosphorylated compounds, microorganisms require active alkaline phosphatases^48^. Because orthophosphates inhibit the activity of phosphatases^49^, in the case of Lake Niegocin, we did not observe the respiration of this type of substrate. We found a negative logarithmic relationship between the P-PO_4_^3−^ concentration and respiration for all lakes we examined. This confirms that the results of the EcoPlate analysis were influenced by inhibitors present in water, making it impossible to precisely characterize the full metabolic potential of the bacteria. At the same time, our data indicate that the EcoPlate test allows the assessment of the current potential of the bacteria. Therefore, the EcoPlate-based metabolic fingerprint should be interpreted as a result of the metabolic capacity of the microorganism community, its taxonomic composition and environmental characteristics (availability of carbon sources, adaptation of bacteria to carbon sources and the presence of inhibitors). Despite these reservations, the Mantel correlation of the Bray-Curtis distance matrices calculated for the community-level physiological profiling distance and taxonomic diversity gave significant results. Although the value of the correlation was rather low (r = 0.22, Mantel test), it remained statistically significant (p = 0.006). The Mantel test results together with the results of the NMPS analysis allow us to conclude that there is some relationship between the taxonomic structure of microorganisms and their ability to decompose the carbon sources in the studied lakes; however, this relationship may be influenced by various environmental factors. Additionally, functional metabolic potentials may be strongly affected not only by bacteria-bacteria interactions and environmental conditions^50^ but also by heterotrophic bacteria-cyanobacteria^36^ or bacteria-eukaryotic algae interactions^51^.

## Conclusions

The Great Masurian Lake System can be considered to be a testing ground that allows us to study the impact of the anthropogenic eutrophication process on the functional and structural diversity of aquatic bacteria. Our results highlight the dependence of the taxonomic composition on lake trophic state and suggest that accelerated anthropogenic eutrophication may lead to a significant transformation of the taxonomic structure of microorganisms from the *Bacteria* domain. This is due to the changes in the physicochemical conditions prevailing in the lakes undergoing anthropopressure, among other reasons. The increase in eutrophication in the studied lakes led to an increase in bacterial family richness and the evenness of their distribution. The change in the taxonomic structure resulting from the change in trophic state did not clearly follow the change in the metabolic structure reflected in the differences in the preference for the use of various carbon sources by microorganisms. The data, however, indicate the existence of a structure-function relationship. Although this relationship is not strong, it reflects the high plasticity of the metabolism of microorganisms depending not only on the taxonomic composition but also on the environmental conditions and, in the context of nutrition, the available resources of labile organic matter. This plasticity is one of the reasons why, despite increasing research, the relationship between bacterial biodiversity and functioning remains poorly understood. The presented study concerns only a small part of the huge population of bacteria living in lakes, mainly the aerobic bacteria inhabiting surface waters of the littoral zone during summer stratification. To what extent the observed relationship between the trophic state of lakes and the functioning of bacteria from the littoral zone is representative of the entire lake bacteriotome requires further research.

## Methods

**Figure 6:**
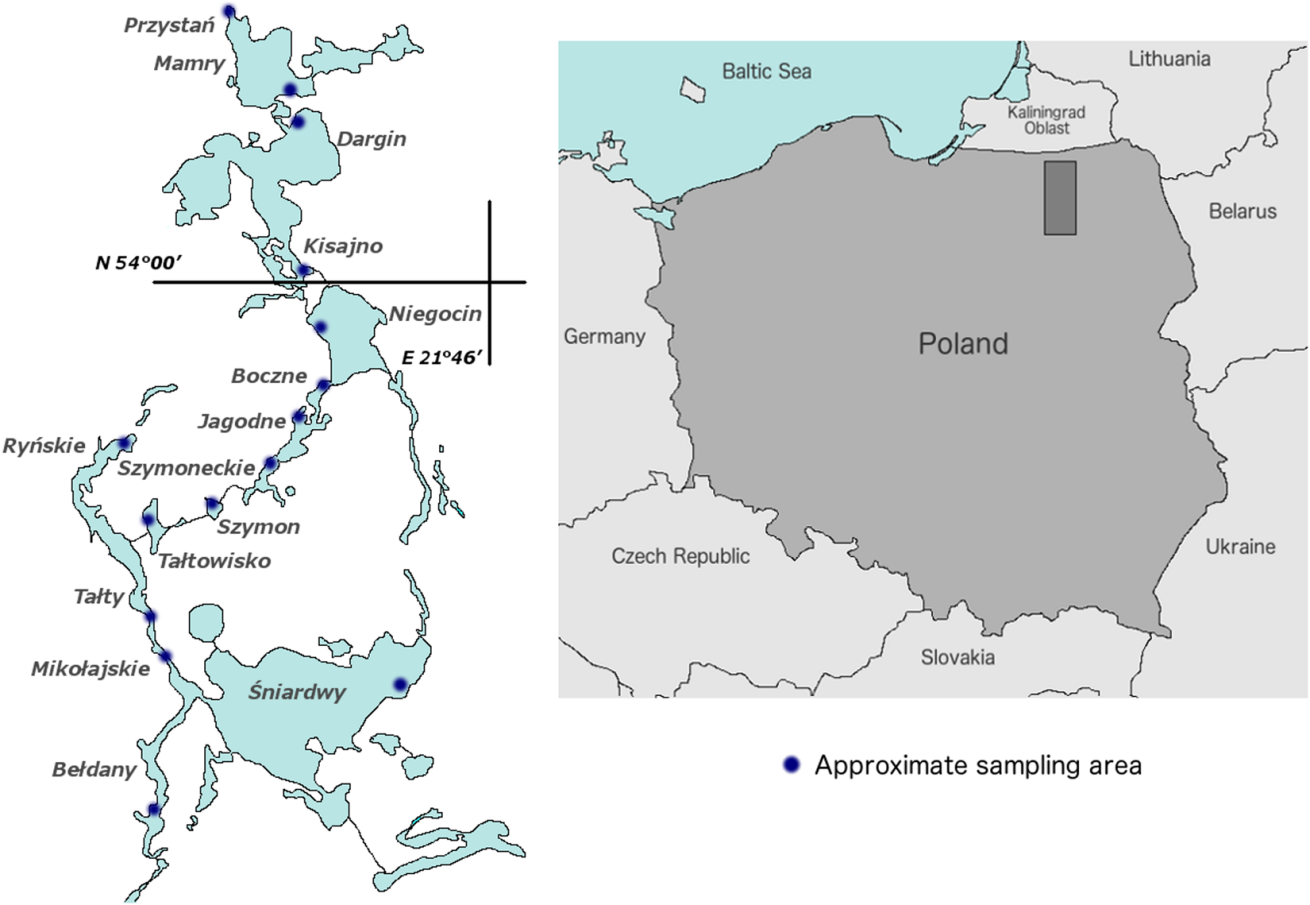
Locations of sampling sites within the Great Masurian Lakes System (GMLS).

The research area comprised 15 lakes belonging to the Great Masurian Lake System (Fig. 6). The GMLS is a chain of lakes located in the Great Masurian Lakes region in north-eastern Poland. All lakes of the GMLS are connected by natural or artificial channels, and create a broad, long chain that is unique in Europe. The northern meso-eutrophic part of the GMLS includes the lakes Przystań, Mamry, Dargin, Łabap, Kisajno and Niegocin, whereas the southern, mostly eutrophic lakes are Boczne, Jagodne, Szymoneckie, Szymon, Kotek, Tałtowisko, Ryńskie, Tałty, Mikołajskie, Bełdany and the meso-eutrophic lake Śniardwy (Fig. 6). Detailed information about the characteristics of the lakes is shown in Table 1. Samples were taken in July 2016 from the surface water of the littoral zones during the summer thermal stratification. The approximate sampling sites are shown in Fig. 6. The samples were taken in 3 repetitions within the radius of 100 m from the central points of sampling area (geographical coordinates in Table 1). Each sample constituted a mixture (v/v) of water sampled from three depths: 1, 2 and 3 metres, taken at all lakes except Mamry, Niegocin, Szymoneckie, Szymon and Śniardwy, where samples were taken at 1 m and 2 m depth because the water was shallower than 3 metres at the sampling sites. The water samples were collected in sterile 5 l polyethylene containers. The samples were subjected to further laboratory analyses within 2–4 h.

The physicochemical parameters (temperature, conductivity, chlorophyll autofluorescence, pH, oxygen concentration, turbidity) were determined *in situ* using a multiparametric YSI 6000 probe (Yellow Spring, YSI Inc./Xylem Inc.USA). Parameters were measured at every 20 cm depth, in each sampling point. The results are presented as the average for the analysed water column. Dissolved organic carbon (DOC) was determined with a Shimadzu TOC 5050 carbon analyser.

The number of DAPI (4,6-diamidino-2-phenylindole) -stainable bacteria was determined by the direct counting of cells collected on 0.2 µm black polycarbonate membrane filters (Millipore) using epifluorescence microscopy^52^. A computer image analysing system composed of a Nikon epifluorescence E450 microscope, Nikon Digital Camera DXM 1200F and NIS elements software (Nikon) were used. HNF (heterotrophic nanoflagellates) were counted under Nikon epifluorescence E450 microscope (Nikon)^53^.

The total phosphorus (TP) and phosphate (PO_4_^3−^) concentrations were determined using the molybdate method using a Shimadzu UV-VIS 1201 spectrophotometer^54^.

Total nitrogen was determined using commercially certified Merck-Millipore cell tests (Spectroquant Nitrogen (Total) Cell Test, 114537) according to the manufacturer’s instructions using Merck Spectroquant Pharo 300 spectrophotometer.

The chlorophyll *a* (Chl a) concentration was determined fluorimetrically using a TD-700 fluorometer (Turner)^55^.

The mean TSI (Trophic State Index) was defined as the arithmetic mean of the TSI calculated on the basis of the concentration of chlorophyll a, total phosphorus and the visibility of the Secchi disc^23^.

### Taxonomic diversity of the bacteria domain

DNA from the samples was isolated after the suspension of microorganisms on the surface of 0.2 µm sterile polycarbonate filters (Merck Millipore). From each lake, exactly 5 × 200 ml of water was filtered on Nalgene (Nalgene®) filtration equipment and immediately frozen in sterile 2 ml Eppendorf vials (each filter separately) at −30 °C. The DNA from the shredded filters was isolated by spin-column-based DNA extraction using the GeneMATRIX DNA Purification Kit (EurX, Gdańsk). The isolated purified DNA was stored at −80 °C for further analysis.

The phylogenetic analysis of the bacterial community was performed using Illumina sequencing^56^. For the sequencing, the 16S rRNA genes, V3–V4 hypervariable regions (amplicons of approximately 459 bp) were selected. PCR amplification was carried out using Q5 Hot Start High-Fidelity 2X Master Mix using reaction conditions as recommended by the manufacturer (95 °C for 3 minutes, 25 cycles of 95 °C for 30 seconds, 55 °C for 30 seconds, 72 °C for 30 seconds and, after the last cycle, 72 °C for 5 minutes) with region-specific (341F and 785R)^57^ primers that include the Illumina flowcell adapter sequences. The primer sequence was as follows: forward primer: 5′ CGGGNGGCWGCAG 3′, reverse primer: 5′ GACTACHVGGGTATCTAATCC 3′. The Illumina overhang adapter sequences added to the locus-specific-sequences were as follows: forward overhang: 5′ TCGTCGGCAGCGTCAGATGTGTATAAGAGACAG (locus-specific sequence), reverse overhang: 5′ GTCTCGTGGGCTCGGAGATGTGTATAAGAGACAG (locus-specific sequence).

Sequencing took place on an Illumina MiSeq sequencer using the MiSeq Reagent Kit v2 and 2 × 250 bp protocol. Initial automatic data analysis was carried out on the MiSeq system using the MiSeq Reporter software (MSR) v2.6, which was used for demultiplexing the data and raw FASTQ file generation. The bioinformatic analysis resulting in the classification of reads up to the species level was carried out using the QIIME software package and the Greengenes v13_8 reference sequence database. The analysis consisted of the following stages:

(1) Removal of adapter sequences and quality filtering (Phred quality score <20, minimum length 30 – Phytonproject – cutadapt); (2) Pair-end read merging (ea-utils in FASTQ-join in QIIME). (3) Clustering – uclust; (4) Chimeric sequence removal – ChimeraSlayer; (5) Taxonomic analysis of OTU clusters – (uclust implemented using the assign_taxonomy.py command in QIIME).

For the Bray-Curtis similarity matrices, the relative abundance of OTUs at the family level expressed as a percentage was used. These values were calculated as the sequence count for each identified group divided by the total sequence counts^58^. For the correlation with TSI, the relative abundance of each group was calculated in the same way at the phylum level.

### Metabolic diversity of microbial communities

The Biolog EcoPlate method was used to determine the community-level physiological profiles^19^ with the modifications described below. EcoPlate (Biolog, USA) is used to measure the ability of the bacterial community to utilize carbon substrates. An EcoPlate is a 96-well microplate composed of triplicates of 31 response wells with different carbon sources and control wells. The following carbon sources were used: carbohydrates—D-cellobiose, α-D-lactose, β-methyl-D-glucoside, D-xylose, erythritol, D-mannitol, N-acetyl-D-glucosamine, D-galactonic acid γ-lactone; phosphorylated carbons—glucose-1-phosphate, D,L-α-glycerol phosphate; amines—phenylethylamine, putrescine; carboxylic acids—D-glucosaminic acid, D-galacturonic acid, γ-hydroxybutyric acid, itaconic acid, α-ketobutyric acid, D-malic acid, pyruvic acid methyl ester, 2-hydroxy benzoic acid, 4-hydroxy benzoic acid; complex carbon—Tween 40, Tween 80, α-cyclodextrin, glycogen; amino acids—L-arginine, L-asparagine, L-phenylalanine, L-serine, L-threonine, glycyl-L-glutamic acid. Each well of a single plate was filled with fresh lake water (150 µl). The plates were incubated in darkness at a temperature of 22 °C for 72 hours. The absorbance was measured every 4 hours at 590 nm wavelength using a Biotek Synergy H1 plate reader (Biotek Corporation, USA). The observed absorbance of tetrazolium chloride reduced due to respiration increased during the incubation time. For analysis, we used the maximal colour development rates (Vmax). For the calculation of Vmax values, Gen 5 software (Biotec Corporation, USA) was used. We identified the slope for every four consecutive reads. Vmax was calculated using a linear regression model by determining the maximum slope during the 72-hour incubation time. The maximal rate of colour development,Vmax (mOD/min), was treated as proportional to the microbial respiration of a single carbon source. To determine the respiration rate of the microbial community without the addition of any carbon substrate, control wells were used.

To determine the influence of each additional carbon source on the respiration rate, the difference in Vmax between carbon-containing wells and control wells was calculated. When the difference (delta Vmax) was higher than zero, we used these results for further analysis. When delta was lower than zero or equal to zero, we assumed that the source was not utilized effectively by the microorganism community, and we set this value of Vmax to zero. For the analyses of community-level physiological fingerprinting, we used the relative values of the respiration rate for each carbon source, calculated as the percentage share of each carbon source in the sum of Vmax for the whole plate.

### Statistical analysis

To group lakes according to their physicochemical parameters, a PCC analysis based on a correlation matrix was used (Statistica, StatSoft, TIBCO Software Inc.).

To group the lakes according to the microorganism metabolic and phylogenetic differences, Bray-Curtis-based NMDS (non-parametric multidimensional scaling) was performed. PAST3 software^59^ was used for the Bray-Curtis similarity matrix calculation. Statistica software (StatSoft, TIBCO Software Inc.) was used for the NMDS analysis and data visualization. To determine whether the differences between eutrophic and meso-eutrophic lakes were statistically significant, the ANOSIM test was used^60^. To assess which taxa were primarily responsible for observed difference between groups, the SIMPER test was used^60^.

For diversity indices, we used richness (number of taxa or used carbon sources) and Simpson’s diversity index (1-*D*) to measure the ‘evenness’ (PAST3 software).

To find the relationship between functional and phylogenetic diversity, the Mantel permutation test was used (PAST3 software). Mantel tests were performed to reveal the correlation between two Bray-Curtis similarity-based matrices of relative metabolic and relative phylogenetic data.

Nonparametric Spearman correlations were calculated using PAST3 software. For table and bar graph preparation, Excel (Microsoft) and Origin (OriginLab) were used.

## Data Availability

All sequencing reads have been deposited at the NCBI Archive - BioProject: PRJNA509870 (https://www.ncbi.nlm.nih.gov/Traces/study/?acc=PRJNA509870). The remaining datasets generated and/or analyzed during the current study are available in the manuscript or from the corresponding author on reasonable request.
